# Emotion recognition and processing in patients with mild cognitive impairment: A systematic review

**DOI:** 10.3389/fpsyg.2022.1044385

**Published:** 2022-10-28

**Authors:** Lucia Morellini, Alessia Izzo, Stefania Rossi, Giorgia Zerboni, Laura Rege-Colet, Martino Ceroni, Elena Biglia, Leonardo Sacco

**Affiliations:** ^1^Faculty of Biomedical Sciences, Università della Svizzera Italiana, Lugano, Switzerland; ^2^Neuropsychological and Speech Therapy Unit, Neurocenter of Southern Switzerland, Ente Ospedaliero Cantonale (EOC), Lugano, Switzerland

**Keywords:** mild cognitive impairment, social cognition, systematic review, tasks, emotion recognition and processing

## Abstract

The purpose of this study was to investigate emotion recognition and processing in patients with mild cognitive impairment (MCI) in order to update the state of current literature on this important but undervalued topic. We identified 15 papers published between 2012 and 2022 that meet the inclusion criteria. Paper search, selection, and extraction followed the PRISMA guidelines. We used a narrative synthesis approach in order to report a summary of the main findings taken from all papers. The results collected are still ambiguous: some studies did not find any differences between MCI and healthy controls (HC) groups in emotion recognition and processing, and other results reported emotion-specific deficits in emotion recognition regarding MCI patients (both regarding negative and neutral emotions). It is essential to underline that these findings could not be generalized to the whole MCI population due to the heterogeneous use of measures and composition of the sample. This does not allow us to make a comprehensive comparison between the results. Our suggestion for future research is to align the results using the same type of tests and emotion recognition assessment.

## Introduction

Emotion recognition and processing are considered the first abilities to establish social interactions (that go through three processes: social perception, social understanding, and social decision-making) (Morese and Palermo, 2020, 2022). Social perception is a fundamental skill of social cognition because the analysis of facial expressions, gestures, posture, language, and voice allows us to recognize others as “living people” (Arioli et al., 2018; Morellini et al., 2022).

In particular, emotion recognition plays an important role in interpersonal communication (Ferretti and Papaleo, 2019). Thanks to the facial expressions of others, it is possible to make inferences about their emotional states. The understanding of emotional cues might lead to a relevant empathic response in emotional situations (Adolphs, 2002; Balconi and Pozzoli, 2009). This ability permits one to anticipate situations, have an adequate response, avoid conflict, and self-regulate emotions (Brüne and Brüne-Cohrs, 2006). Deficits in this domain could contribute to several problems in daily life, such as difficulties in interpersonal relationships (Palermo et al., 2020); they can also predict psychiatric or neurodegenerative disorders (Ferretti and Papaleo, 2019). As an example, weaker emotion recognition might influence social behavior (Mueser et al., 1996) that could become inappropriate. Also, this type of impairment is correlated with higher levels of depression (Chiu et al., 2006), interpersonal difficulties, social isolation, and psychobehavioral disturbances (Shimokawa et al., 2001) that could lead to an increased risk of institutionalization (Brodaty, 1996).

Till present, we do not know how old people’s pathologies affect emotional processing and social cognition in general (Cárdenas et al., 2021).

However, the vulnerability in social cognition mechanisms is age-related, as reported in the literature (Williams et al., 2009); in particular, evidence shows that younger adults have better performance than older adults in recognizing basic emotions (McCade et al., 2011). In addition, emotion recognition can be more impaired in older adults with disorders that involve social cognition’s brain structure, such as MCI or other dementias (Amlerova et al., 2022).

Mild cognitive impairment is considered a prodromal phase of dementia, a transitional stage between healthy aging and dementia (Anderson, 2019; Breton et al., 2019). The risk of developing dementia in the following years is higher in this specific phase, but their daily functioning is still mainly preserved (Petersen, 1996; Smith et al., 1996). The first definition of MCI belongs to Petersen et al. (1997), which describes a moment of transition where patients, as reported before, do not meet the dementia’s criteria.

In this regard, MCI is also described by the DSM 5 (American Psychiatric Association, 2013) through a diagnostic category named “mild cognitive disorder (mild-NCD),” which fits with Petersen et al. (1997) and Albert et al. (2011) criteria. These diagnostic criteria highlight a mild cognitive decline in one or more domains (for example, language and memory); it also reports that the decline does not interfere with daily routine and cannot be explicated by mental disorders or delirium (for example, schizophrenia or depression) (American Psychiatric Association, 2013).

Petersen (2016) delineated an important distinction between two different types of MCI, namely, amnestic MCI (aMCI) and non-amnestic MCI (naMCI). The first one considers deficits only in the memory domain. In contrast, the naMCI is related to single or multiple cognitive domain impairments, such as visuospatial abilities, language, memory, and executive functions.

Currently, there are several studies on the topic, but the last review performed refers to 10 years ago (McCade et al., 2011), and their results were ambiguous. Based on that, we decided to present a new systematic review describing the literature of last decade.

The aim of this study was to investigate emotion recognition and processing in MCI patients to update the state of current literature on this crucial but underestimated topic.

We took into account only results about this social cognition domain because our purpose was to understand the strengths and weaknesses of this argument in the current literature. To achieve this goal, considering that the selected articles were enough/adequate to conduct a systematic review, we found it necessary to focus on a single domain.

### The goal of this study review

The aim of this study is led by the need to understand and update the state of the current literature regarding emotion recognition and processing in MCI. To date, the last review on this topic was made by McCade et al. (2011), and their results are ambiguous; that is the reason behind the choice to collect and analyze the previous findings on this field.

Based on the past research on social cognition in neurodegenerative disorders (Morese and Palermo, 2020; Palermo et al., 2020; Morellini et al., 2022), we could expect that emotion recognition and processing might be impaired in the MCI population.

## Method

This systematic review was conducted according to the Preferred Reporting Items for Systematic Reviews and Meta-Analyses (PRISMA, Moher et al., 2009). The method is currently available in the Open Science Framework (OSF).^1^

### Eligibility criteria

The aim of this systematic review was to gather studies that analyze the functionality of emotion recognition and processing in patients with mild cognitive impairments (MCI).

The inclusion criteria were the following:

• Studies with a population with a diagnosis of mild cognitive impairment (MCI), evaluated by standardized diagnostic criteria of Petersen (1996, 2004), Petersen and Negash (2008), Petersen (2016) or DMS 5—DSM IV—DSM IV-TR criteria (American Psychiatric Association, 1994, 2000, 2013) or Winblad et al. (2004) or Albert (PD-MCI—Albert et al., 2011) or every cognitive impairment—without dementia—diagnosed with a validated cognitive test; for example, Dementia Rating Scale (DSR) (Mattis, 1988) or PD-MCI (Litvan et al., 2011).
• Study sample must be over 60 years old, either male or female.
• All types of MCI: amnesic MCI (aMCI), non-amnesic MCI (naMCI), Parkinson MCI (PD-MCI), Alzheimer MCI (AD-MCI), and Vascular MCI (VaMCI).
• Studies that evaluated the domains of social cognition, “emotion recognition and processing.”
• Studies must include at least one clinical cognitive measurement for the social cognition domain analyzed (emotion recognition and processing).

The exclusion criteria were the following:

• Articles not in English were excluded.
• Meta-analysis, systematic reviews, single case studies or other studies with a small sample (i.e., studies with less than 10 participants) or only qualitative measurements, comments, books, conference papers, letters, theses, and all other studies not peer-reviewed were excluded.

## Information sources

### Search strategy

The search for this study was conducted across PubMed and Medline databases. For the MCI search strategy, we used the terms “MCI” OR “mild cognitive impairment.” The keywords were combined with the domain of social cognition “emotion recognition and processing,” to produce the results.

### Study selection

We only considered studies that are limited to humans, with a limited range of periods from January 2012 to May 2022. We considered the studies in the past 10 years because of the number of papers and because they are appropriate for our time and resources. Moreover, meta-analysis, other systematic reviews, case studies, qualitative studies, or every study with a very small sample without quantitative measurements were excluded from this review. Initially, 256 papers were included in the selection and then 53 duplicates were excluded. Reading the title and abstract of 203 articles, we excluded 166 other articles from the topic. Only 37 papers were considered eligible for the scope of this review. Those 37 papers were further analyzed by reading the complete text to discover if they met the inclusion criteria. At this point, the other 22 articles were excluded because 17 did not have an MCI diagnosis (in line with inclusion criteria), one did not investigate the domain of social cognition elected for this review, and the last 4 were reviews or meta-analyses (as described previously in the inclusion criteria).

In the end, 15 articles were included in our review.

**FIGURE 1:**
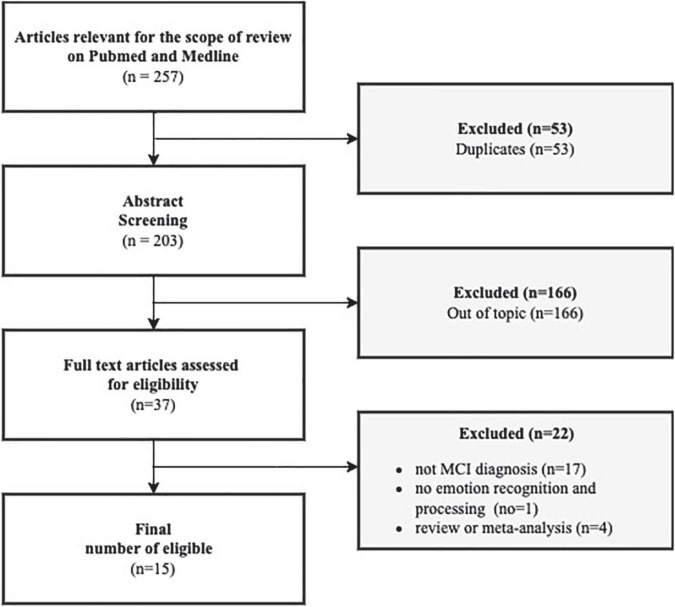
Flow diagram.

Despite the age range (2012–2022), we found only eligible articles until 2020 (Figure 1).

## Results

Overall, 15 studies were included in our review. Those studies examine the domain of social cognition and emotion recognition and processing. We examined articles from 2012 to 2022, but we found eligible articles only until 2020.

### Participant characteristics

In the 15 studies included in our review, participant ages ranged from 63.70 (McCade et al., 2013) to 79.9 (Sapey-Triomphe et al., 2015).

The sample size varied from a minimum of 10 MCI (Bediou et al., 2012; Parra et al., 2013) to a maximum of 92 MCI (Hayashi et al., 2021); most of the studies differentiated MCI types (for example, aMCI, naMCI, single/multiple domain MCI) (Bediou et al., 2012; Richard-Mornas et al., 2012; McCade et al., 2013, 2018; Sapey-Triomphe et al., 2015; Schefter et al., 2013; Varjassyová et al., 2013; Yang et al., 2015; García-Casal et al., 2019).

### Diagnostic criteria

The main diagnostic criteria adopted to assess MCI patients were described by Petersen et al. (1997, 1999), Petersen (2004) and Petersen and Negash (2008), which were used in most of the studies in this review (Bediou et al., 2012; Henry et al., 2012; Richard-Mornas et al., 2012; McCade et al., 2013, 2018; Varjassyová et al., 2013; Pernigo et al., 2015; Sarabia-Cobo et al., 2015; Yang et al., 2015; Park et al., 2017).

Other studies adopted Winblad et al. (2004) criteria (Henry et al., 2012; Schefter et al., 2013), Albert et al. (2011) criteria were used by Parra et al. (2013), the Clinical Dementia Rating (CDR, Hughes et al., 1982) was used by Sapey-Triomphe et al. (2015), and DSM-IV/DSM-IV-TR/DSM-5 (American Psychiatric Association, 1994, 2000, 2013) was adopted by Park et al. (2017), García-Casal et al. (2019), and Hayashi et al. (2021).

### Emotion recognition and processing measures

The methodologies used to assess emotion recognition and processing were various. All the studies adopted different types of tasks.

The Facial Expression Recognition Task (FER; Ekman and Friesen, 1976) was the test most used by the authors (Bediou et al., 2012; Richard-Mornas et al., 2012; Varjassyová et al., 2013; Sapey-Triomphe et al., 2015; Hayashi et al., 2021), and the Gender Recognition Task (Bediou et al., 2009) was endorsed by Bediou et al. (2012) and Sapey-Triomphe et al. (2015).

Measures adopted by the other authors are described in Table 1 due to the large number of methodologies selected for each study included in our review.

**TABLE 1:** Emotion recognition measures.

References	Name	Description
Ekman and Friesen, 1976	Facial Expression Recognition Test (FER)	This test includes pictures from the Ekman and Friesen series used to measure facial emotion recognition. Those pictures represent the six basic emotions (happiness, anger, sadness, fear, surprise, and disgust) and a random description is written under each image. Every face displays four different emotion. The aim of the task is to name or point out the emotion which match better with the facial expression shown in the pictures.
Bediou et al., 2009	Gender recognition task	This test includes face’s photos of eight males and eight females of average age. Every faces presented have a neutral expression. This test is a forced-choice paradigm, so after each face displayed, participants should indicate the gender of the face (man/woman).
Vanrie and Verfaillie, 2004	Biological motion task	Twenty-four point light animations divided in 2 blocks: 12 pictures of action (which were crawling, cycling, drinking, driving, jumping, playing pool, a tennis serve, rowing, saluting, sawing, sweeping, and digging) and 12 pictures which figure out the 6 basic emotions. For the action variant, participants have to make a response by their own, for the emotion variant, the test showed the labels with the six6 basic emotion within choice.
Ekman and Friesen, 1976	Pictures of facial affect	This task includes 36 black and white photographs from the set of Ekman and Friesen’s Pictures of Facial Affect.
Benton et al., 1994	Short Form Benton Facial Recognition Test (BFRT)	BFRT shows 13 still black and white photos of unknown male and female faces. The assignment of this task is to match the photograph proposed with an identical another one or with another three photographs which represent the proposed one taken from different angles. This task has no time limit.
Young et al., 2002	Facial expression of emotion: stimuli and test (FEEST) Ekman 60 faces test	FEEST is a computerized task taken from the Facial Expressions of Emotion: Stimuli and TestsCD-ROM (FEEST; Young et al., 2002). Includes 60 black and white still photographs from the Ekman and Frieses set, which includes the six basic emotions. Every photographs are presented for 5 s in a randomized order on a computer screen with the six labels of the basic emotion displayed below. Is an emotion recognition task with prompts.
Tottenham et al., 2009	NIMSTIM set of facial expression	This test in an emotion identification task. It contained pictures that represent expressions of disgust, fear, happiness, anger, sadness, and surprise, as well as a neutral pose. The photographs are displayed for 4 s on a computer screen and participants have to respond to a question like “how is this person feeling?” in order to identify emotional representation showed by the actors. Is an emotion recognition task without prompts.
Losh et al., 2009	Movie still task	This test asks participants to determine the emotional contents of complex scene and investigate the ability of each individual to use facial and body informations in order to complete the task.
Lang et al., 1988; Parra et al., 2013	Emotional memory paradigm	This test is composed by 72 images that were selected (according to their valence and arousal) from the International Affective Picture System (Lang et al., 1988). They comprehend: -36 images depicting emotionally neutral scenes -36 depicting emotionally positive scenes.
Schefter et al., 2013	248 portraits of unknown faces	In this selection of portrait [taken from five different sources (Matsumoto and Ekman, 1988; Lundqvist et al., 1998; Martinez and Benavente, 1998; Tottenham et al., 2009)] half of them displayed neutral expressions and half angry expressions.
Keane et al., 2002	Identification of Pictures of Famous Person (FFI) adapted in the studies for Czech personalities (Bechynì et al., 2008).	FFI task proposes 10 pictures of famous people and participants were asked to decide if the person was familiar/unfamiliar.
Pernigo et al., 2015	Emotional discrimination task	This task is composed by four categories of stimuli representing: Humans (bodies): body stimuli were black and white pictures representing a male subject. Humans (faces): face stimuli were a selection of the neutral and fearful facial expressions of the Ekman and Friesen set (Ekman and Friesen, 1976). Living non-humans (dog faces). Inanimate objects (knives). In this task the sample and the stimuli had different emotional contents, but portrayed the same model.
Pernigo et al., 2015	Identity discrimination task	This task is composed by four categories of stimuli representing: Humans (bodies): body stimuli were black and white pictures representing a male subject. Humans (faces): face stimuli were a selection of the neutral and fearful facial expressions of the Ekman and Friesen set (Ekman and Friesen, 1976). Living non-humans (dog faces). Inanimate objects (knives). In this task the sample and the stimuli, contrary to the previously reported, has the same emotional content but the model various from identity/morphology (such as, a dog or a knives).
Sarabia-Cobo et al., 2015	Emotional Facial Expression (EFE)	This test is a forced choice task, designed to assess emotional processing. Participants had to identify the emotion expressed by a face, choosing one of six possible answers: happiness, sadness, anger, surprise, fear, or disgust [Pictures taken from Ekman and Friesen (1978)]
Yang et al., 2015	China faces emotions materials database	This test is composed by a selection of 300 pictures from the Chinese database of Wang and Luo (2005). The pictures can be divided in: 100 positive faces 100 neutral faces 100 negative faces. The pictures were also divided between men and women.
García-Casal et al., 2019	Affect-GRADIOR	This test is a computer-based emotion recognition test in older adults with or without cognitive impairment. The pictures comprehend emotional stimuli (six basic emotions and a neutral expression) through color photographs of professional actors.

It is necessary to point out that two studies out of 15 (Parra et al., 2013; Yang et al., 2015) included neuroimaging measures: fMRI was used to investigate the emotional memory associated with emotion recognition, which could be deteriorated in patients with MCI. EEG was registered to investigate the features of emotional face processing using event-related potentials (ERPs).

### Summary of relevant findings

All the articles described in our review investigated emotion recognition and processing in patients with MCI; it’s necessary to underline that the results obtained differ due to the variety of tasks adopted by the authors and the sample’s composition.

In this review, eight studies out of 15 reported homogeneous results (Bediou et al., 2012; Richard-Mornas et al., 2012; Sarabia-Cobo et al., 2015; Yang et al., 2015; Park et al., 2017; McCade et al., 2018; García-Casal et al., 2019; Hayashi et al., 2021). Specifically, 4 of them showed similar performances in emotion recognition and processing between MCI patients and healthy subjects: Bediou et al. (2012) found out that patients with aMCI (*N* = 10) showed no deficits compared with the HC group in either of the two tests adopted (refer to Table 2). Correspondingly, Park et al. (2017) reported that the performance of MCI patients (*N* = 32) was not significantly different compared to the HC group. On the same line, McCade et al. (2018), who evaluated the visual processing of emotional faces by using two different tests (refer to Table 2), obtained the same results: the performances of aMCI (*N* = 14) and naMCI (*N* = 18) were similar to the HC group. The most recent study, conducted by Hayashi et al. (2021), followed the same line and found that the MCI participants (*n* = 92) did not differ in feelings of anger and sadness in comparison with the HC group.

**TABLE 2:** Emotion recognition (*N* = 15).

References	Participants	Mean Age (MCI sample)	Summary of relevant findings	Cognitive measures adopted
Bediou et al., 2012	10 = HC 10 = aMCI [Petersen et al. (1999) criteria]	73	Gender recognition was preserved in every condition. MCI patients didn’t show any deficit compared to HC group (The only difference was for the PD group in FER)	Facial Expression Recognition Test (FER) Gender recognition task
Henry et al., 2012	38 = HC 36 = MCI [Winblad et al. (2004) criteria; Petersen et al. (1999) criteria]	78.3	MCI group did not differ from HC group in the Biological Motion Task, despite showing difficulties with decoding of Facial Emotion.	Biological motion task Pictures of facial affect
Richard-Mornas et al., 2012	17 = HC 12 = aMCI [Petersen et al. (1999) criteria]	68.5	aMCI group performed worse than HC in fearful facial expression, mainly when the gaze stimulus is not presented and there is not direct attention to the eye region.	Short Form Benton Facial Recognition Test (BFRT)
McCade et al., 2013	17 = HC 16 = naMCI; 19 = aMCI [Petersen (2004) criteria]	63.78–69.63	There were no significant differences between HC and MCI group in the *BFRT*. aMCI group showed significant differences in the recognition of anger (*FEEST*), while naMCI showed the similar results of HC group. Regarding the *Emotion Identification Task*, aMCI performed worse in the recognition of anger, while they did not differ in neutral faces. naMCI group did not show significant differences on any measure of emotion recognition compared to HC. aMCI patients performed worse in the *Movie Stills Task* then HC group in recognizing total emotions with or without facial cues. On the other hand, naMCI group showed no significant differences from HC.	Short Form Benton Facial Recognition Test (BFRT) Facial Expression of Emotion: Stimuli and tests (FEEST) NimStim set of facial expression Movie Stills Task
Parra et al., 2013	10 = HC 10 = MCI [Albert et al. (2011) criteria]	76	MCI patients showed behavioral impairment in the Emotional Memory Paradigm, but they did not show neuroimaging changes	Emotional memory paradigm
Schefter et al., 2013	17 = HC 15 = aMCI [Winblad et al. (2004) criteria]	67.13	Results showed that, compared to HC, aMCI group recognition of negative faces was preserved, while recognition of neutral faces was impaired.	248 portraits of unknown faces
Varjassyová et al., 2013	18 = HC 10 = SD-aMCI; 10 = MD-aMCI [Petersen (2004) criteria]	74.0–77.8	Regarding the aMCI group, there were significant deficits in FER but not in FFI	Facial Expression Recognition Task (FER) Identification of Pictures of Famous Person (FFI)
Pernigo et al., 2015	24 = HC 24 = MCI (Mayo Criteria, Petersen and Negash, 2008)	74.4	MCI group and HC group performed similar in the *Identity Discrimination Task*, while MCI group had a worse performance in the *Emotional Discrimination Task*. In the *BEES*, *TAS-20*, and *IRI* there were not found significant differences (except for the Perspective Taking subscale of IRI).	Emotional discrimination task Identity discrimination task
Sapey-Triomphe et al., 2015	39 = HC 15 = aMCI (Clinical Dementia Rating—CDR, Hughes et al., 1982)	79.9	The two groups performed worse than HC in the *FER*. They showed similar performances regarding the recognition of neutral emotions. The recognition of anger, fear, disgust, and happiness was the most impaired. For the Gender Recognition Task, there were not significant differences between all groups.	Facial gender recognition task Facial Expression Recognition Test (FER)
Sarabia-Cobo et al., 2015	50 = HC 50 = MCI [Petersen et al. (1999) criteria]	77.86	HC group performed better in all the tests than MCI group.	Emotional Facial Expression (EFE)
Yang et al., 2015	24 = HC 24 = aMCI [Petersen (2004) criteria]	71.50	aMCI patients’ accuracy of face recognition memory was lower than HC group. No differences were underline between emotional categories.	China faces emotions materials database
Park et al., 2017	33 = HC 32 = MCI (DSM IV criteria–American Psychiatric Association, 1994; Petersen et al., 1997 criteria)	74.34	MCI’s performance of FER weren’t significant compared to HC.	Facial Emotion Recognition Test (FER)
García-Casal et al., 2019	69 = HC 59 = aMCI (DSM IV-TR criteria; American Psychiatric Association, 2000)	77.60	Results showed that aMCI patients had poorer performances than HC.	Affect-GRADIOR (touchscreen Emotion Recognition Test)
McCade et al., 2018	18 = HC 18 = naMCI; 14 = aMCI [Petersen (2004) criteria]	63.8–67.9	naMCI and aMCI do not differ from HC groups regarding gaze strategies while processing emotional faces (the visual processing of emotional faces was similar in all groups).	NimStim set of facial expression Ekman 60 Faces Test (FEEST)
Hayashi et al., 2021	32 = HC 92 = MCI (DSM 5 criteria; American Psychiatric Association, 2013)	75.7	MCI and HC groups did not differ in the recognition of anger and sadness	Facial Emotion Recognition Test (FER)

On the contrary, four further studies underline the worse performance of MCI patients compared to the HC group in emotion recognition tasks. For example, Richard-Mornas et al. (2012) reported worse performances in fearful facial expressions in patients with aMCI (*N* = 12) compared to the control group. In line with this finding, a study by Sarabia-Cobo et al. (2015) showed the same results (refer to Table 2). Finally, two studies (Yang et al., 2015; García-Casal et al., 2019) reported lower accuracy and poorer performance in face recognition in patients with aMCI (*N* = 24).

The remaining studies reported ambiguous results. For instance, Henry et al. (2012) discovered that MCI patients did not report performance differences in the Biological Motion Tasks (Vanrie and Verfaillie, 2004), while there were remarkable difficulties with decoding facial emotions (Ekman and Friesen, 1976). Likely, Schefter et al. (2013) adopted only one test (refer to Table 2), from which they obtained two different results: aMCI group (*N* = 15) displayed deteriorated performance in the recognition of neutral faces, while the recognition of negative faces was preserved.

Another study (Pernigo et al., 2015) assessed emotion recognition and processing with two different tasks [Emotional Discrimination Task (Pernigo et al., 2015), and Identity Discrimination Task (Pernigo et al., 2015)] on a sample of 24 patients with MCI. Identity Discrimination Task obtained similar results between the two groups. Meanwhile, the Emotional Discrimination Task showed a worse performance for MCI patients.

The research of Varjassyová et al. (2013) compared MCI single and cognitive domains (SD-aMCI, *N* = 10; MD-aMCI, *N* = 10) and adopted two different tasks to assess emotion recognition (FER and FFI, refer to Table 2). Both aMCI groups reported significant deficits in FER but not in FFI, and the impairment was more significant for MD-aMCI.

Two studies reported emotion-specific deficits (McCade et al., 2013; Sapey-Triomphe et al., 2015); in particular, Sapey-Triomphe et al. (2015) used a large battery of tests (refer to Table 2) and found that aMCI group (*N* = 15) performed worse than healthy subjects in the FER (Ekman and Friesen, 1976). The group showed similar performances regarding the recognition of neutral emotions, while the recognition of anger, fear, disgust, and happiness was the most impaired. At the same time, there were no significant differences for the Gender Recognition Task (Bediou et al., 2009), in line with the results mentioned before, reported by Bediou et al. (2012).

On the equal line, McCade et al. (2013), using an extensive battery of tests, showed that patients with aMCI (*N* = 19) performed worse in emotion recognition, especially regarding the recognition of anger, while they did not report significant differences in neutral faces. On the contrary, naMCI (*N* = 50) group did not reveal any deficit compared to HC.

In the end, the neuropsychological studies underline more accurate results: Yang et al. (2015) investigated the time course of emotional face recognition in patients with aMCI (*N* = 24) through the use of EEG and ERP data. The results showed that the aMCI and HC groups obtained similar results in visual processing (ERP P100). Nonetheless, in accordance with the cognitive results, the face structural encoding (ERP N170) was delayed in patients with aMCI.

In Parra et al.’s (2013) study, cognitive results showed impairment in the recognition of emotion in MCI patients (*N* = 10), but there were no neuroimaging changes.

## Discussion

This review had the aim of investigating emotion recognition and processing in patients with MCI in order to update the state of current literature on this important but undervalued topic.

There is another important consequence of these studies’ analysis to highlight: knowing which types of cognitive impairments are associated with neurodegenerative disorders could have an important predictive role. Also, knowing the emotional situation (in this case, emotion recognition and processing) could help to lay out a more detailed cognitive profile, which might permit clinicians to determine effective strategies and programs of prevention.

Nevertheless, from the results reported previously, we did not find aligned outcomes. In this section, we try to explore the reasons behind them. The ambiguous results make it difficult to conclude: most of the studies reported cross-over results (Henry et al., 2012; McCade et al., 2013; Parra et al., 2013; Schefter et al., 2013; Varjassyová et al., 2013; Pernigo et al., 2015; Sapey-Triomphe et al., 2015). On the contrary, some of the studies obtained homogeneous results: a part of them showed MCI’s worse performances on emotion recognition and processing tasks (Richard-Mornas et al., 2012; Sarabia-Cobo et al., 2015; Yang et al., 2015; García-Casal et al., 2019). Meanwhile, the remaining studies reported similar performances between the MCI and HC groups (Bediou et al., 2012; Park et al., 2017; McCade et al., 2018; Hayashi et al., 2021).

Those results could have several underlying reasons. First, the wide selection of the tasks adopted by the authors makes it difficult to compare and overlap the results obtained. This heterogeneity could also be considered a strength because it allows us to explore the emotion recognition domain comprehensively. However, the recommendations for the future research are to achieve unanimity regarding the use of all the tests in order to give more validity to the results.

Despite the various methodologies, we underline a common ground: Some studies reported an emotion-specific deficit in MCI patients (those results concern every type of MCI). The emotion-specific deficits regard the recognition of negative emotions (especially for fear and anger), as shown by Richard-Mornas et al. (2012), McCade et al. (2013), and Sapey-Triomphe et al. (2015), while the recognition of neutral emotions seems to be unimpaired, except for the findings of Schefter et al. (2013), which reported opposing results.

Another aspect to highlight is the sample composition that, in the studies included in our review, varied from a minimum of 10 (Bediou et al., 2012; Parra et al., 2013) to a maximum of 92 (Hayashi et al., 2021). This might be why it has been arduous to overlap the full results and, at the same time, could involve a wrong generalization and overestimation of the findings. Despite everything, we decided anyway to include studies with a small sample because the research was limited and we wanted to get a complete picture of this topic.

In addition, studies had a different sample composition based on the ways to diagnose MCI (i.e., DSM IV; American Psychiatric Association, 1994 or Petersen, 1996). For instance, some studies have a sample of patients with aMCI only (Bediou et al., 2012; Richard-Mornas et al., 2012; Schefter et al., 2013; Varjassyová et al., 2013; Sapey-Triomphe et al., 2015; Yang et al., 2015; García-Casal et al., 2019); others have included both types of aMCI and naMCI subjects (McCade et al., 2013, 2018).

Against this background, a good suggestion for future research could be to divide MCI patients based on their categorization in order to make an equal comparison of their results.

Finally, it would be empowering to conduct longitudinal studies to investigate the association between the impairment of emotion recognition and neurodegeneration.

For instance, it could be interesting to see if social cognition deficits, in particular in emotion recognition, deepen simultaneously with the MCI course. This hypothetic exploration could lead to many scenarios, such as the deterioration of this ability over time, the impact of the etiology on this ability impairment (for example, AD-MCI or PD-MCI) or if social cognition impairment could be predictive of major neurodegenerative disorders.

Finally, regarding the neuropsychological studies, the current findings on this topic are very poor.

We highlight the necessity to adopt more of this methodology because it provides more accuracy and precision in results. For example, thanks to the study by Yang et al. (2015), we know that the time course of emotional face recognition (assessed with EEG and ERP data) could lend different results depending on the stage considered (i.e., in MCI patients, the results in the visual processing are similar compared to those in HC, instead of the results in the structural encoding of the face that are worse).

It would be helpful and functional to adopt a combined analysis (cognitive and neuropsychologic assessment) that could better equip the scientific community with more objective and accurate data. These latter might have a more predicting power to assess MCI and address future diagnoses.

## Conclusion

This systematic review analyzed the literature of last decade on emotion recognition and processing in MCI population. On the database, there is another review on the topic (McCade et al., 2011), but it dates from 2011, so that is the reason behind the choice to explore this field and obtain new findings.

Regarding 2011, the papers eligible for this work were more than twice (6 in 2015 in 2022), but the results are still ambiguous: heterogeneity measures, different size samples, and ways to diagnose MCI patients do not allow us to align and compare the studies in order to widen the results. At the same time, we could highlight a common point: some studies showed that negative emotions are the most difficult to recognize and process, so there is an emotion-specific deficit (Richard-Mornas et al., 2012; McCade et al., 2013; Sapey-Triomphe et al., 2015). Schefter et al. (2013) underlined that there is also an emotion-specific deficit, but in recognizing neutral emotions.

As mentioned before, different samples and measurements could be a limit to generalizing, while this wide range of options could permit the scientific community to understand and address toward the most suitable methodology design.

In conclusion, neuropsychological approach is still almost unexplored (just 2 studies out of 15), so this might be an encouragement to further explore this path.

## Data availability statement

The original contributions presented in this study are included in the article/supplementary material, further inquiries can be directed to the corresponding author.

## Author contributions

LS carried out part of the literature search, collected part of the studies, and reviewed the final version manuscript. LM had the main contribution in the literature search and selection, created the draft, two tables, and flow diagram, dealt with defining and writing the method, wrote the abstract, introduction, part of the results, discussion, conclusion, reviewed the references, and final version of the manuscript. AI elaborated Table 1, wrote part of the introduction, results, discussion, and conclusion, wrote the abstract, reviewed the references, and final version of the manuscript. SR, GZ, and EB contributed to the collected part of the literature search. MC and LR-C contributed to the collection and selection of the literature. All authors contributed to the article and approved the submitted version.

## Footnotes

1
https://osf.io/hn7mc/
